# New Perspectives in Transcranial Magnetic Stimulation: Epilepsy, Consciousness and the Perturbational Approach

**DOI:** 10.3233/BEN-2012-120263

**Published:** 2013

**Authors:** Paolo Manganotti, Alessandra Del Felice

**Affiliations:** Department of NeurologicalNeurophysiologicalMorphological and Motor SciencesUniversity of VeronaVeronaItaly

**Keywords:** TMS-EEG co-registration, cortical excitability, sleep deprivation, sleep

## Abstract

Transcranial magnetic stimulation (TMS) evolved from a simple method to stimulate the motor cortex to an invaluable tool for multiple diagnostic, research, and therapeutic applications. A further development of this noninvasive brain stimulation technique is concomitant electroencephalographic (EEG) recording during TMS. The theoretical underpinnings and the technological innovation of TMS-EEG co-registration have opened new ways to study brain excitability in neurological conditions previously investigated with conventional EEG alone.

A further advance in TMS research applications is the perturbational approach: magnetic pulses can interfere not only with dynamic, often pathological rhythms in epilepsy or altered consciousness states, but also modulate physiological states such as sleep and sleep deprivation. So applied, TMS-EEG co-registration can reveal different neurophysiological and behavioral patterns in the awake state, sleep or sleep deprivation.

In this review, we discuss the use of TMS and TMS-EEG co-registration in epilepsy, a still rather limited although promising area of study.

## 1. Standard TMS parameters and the novelty of TMS-EEG co-registration in epilepsy

Transcranial magnetic stimulation (TMS) is an intriguing tool for studying epilepsy. Simple to perform and relatively inexpensive, it relies on several noninvasive parameters for measuring brain excitability in seizures disorders [1]. Electroencephalography (EEG), which has traditionally been considered as the standard clinical neurophysiological method to investigate and define epileptic disorders, measures the extracellular current flow of excitatory postsynaptic potential (EPSP). With TMS several indexes of measurement of brain excitability can be obtained. TMS parameters of cortical excitability depend on the stimulation paradigm (single pulse or paired pulse). Single-pulse TMS paradigm evaluates the motor threshold (MT), the motor evoked response amplitude and the cortical silent period (CSP), whereas short intracortical inhibition (SICI), short intracortical facilitation (SICF) and long intracortical inhibition (LICI) are investigated with paired-pulse TMS.

The motor threshold is the minimal threshold intensity needed to obtain a motor response. Given a stable spinal motor excitability, MT is thought to represent a measure of pyramidal neuron membrane excitability [2]. Progressive increments in pulse intensity generate a recruitment curve: the resulting modulation of motor evoked potential (MEP) amplitude to an increasing intensity of TMS pulses provides a measure of excitatory feedback to corticospinal output, chiefly mediated by glutamate [3,4].

ISSN 0953-4180/13/$27.50 © 2013 – IOS Press and the authors. All rights reserved

The cortical silent period (CSP) is the lapse of the electromyogram (EMG) when single-pulse TMS is given during a tonic voluntary contraction and it represents GABAergic inhibition at different levels of the motor pathways [5]. The first is the spinal level, with a reduction in muscle fiber spindles discharge and a concomitant activation of inhibitory Renshaw's cells [6]; the second is the supraspinal level that constitutes the second part of the silent period and reflects the activation of inhibitory GABAB interneurons [7,8].

Different paired-pulse protocols yield different parameters such as SICI, SICF and LICI.

Short intracortical inhibition (SICI) consists of a first conditioning subthreshold stimulus applied 2 to 5 msec before the suprathreshold second test stimulus [9]. The first subthreshold stimulus is believed to activate low-threshold cortical inhibitory circuits which inhibit the action potential generated by excitatory postsynaptic potentials (EPSP) induced by the suprathreshold second pulse. Attention should be paid when applying this protocol, since with an interval of 2–3 msec and the first stimulus at 95% of active MT a consistent contamination by SICF is apparently recorded [10]. Nonetheless, this parameter likely reflects GABAA receptor-mediated inhibition, as demonstrated in pharmacological studies by Di Lazzaro et al. [11,12], Ziemann et al. [13], and Florian et al. [14].

Interestingly, a decrease in SICI has been demonstrated after sleep deprivation [15] but not during sleep itself [16,17] presumably because of the GABA imbalance known to occur in a sleep-deprived condition, while an age-dependent relationship has recently been demonstrated with a SICI and LICI increase in older individuals owing to a GABA imbalance [18]. Moreover, increasing test TMS intensities resulted in a progressive reduction in the estimate of SICI, so that estimates of SICI are systematically affected by the intensity of the test TMS pulse, regardless of the excitability state [19].

Intracortical facilitation (ICF) paradigms have a paradigm similar to SICI but with slightly longer (6–20 ms) interstimulus intervals. Its physiology is not entirely clear, and it is thought to excite excitatory neurons of the motor cortex, with a net facilitatory effect derived from a balance between strong NMDA-mediated facilitation [20,21] and weaker GABAA-mediated inhibition [22,23].

Short intracortical facilitation (SICF) differs from both SICI and ICF: the first stimulus is a suprathreshold and the second is a subthreshold [24], or both pulses are approximately of threshold intensity [25]. The observation that SICF occurs at intervals of about 1.5 msec led to the hypothesis of a common generator of SICF and I-waves: the second pulse would excite the axons of the excitatory interneurons depolarised by the first EPSP but that did not yet fire [26]. GABAA agonists reduce SICF [24].

Still longer interstimulus (50–300 ms) interval paired-pulse TMS-EMG protocols measure GABAB receptor-mediated long-interval intracortical inhibition (LICI) [27], reducing the MEP amplitude by 50% of the stimulus applied alone [28], which is enhanced by the GABAB receptor agonists [29,30] and baclofen [29, 31].

Furthermore, adjunctive collision techniques applied to study the integration of different systems, such as sensory motor integration [32–35], employ a peripheral sensory stimulus preceding the TMS pulse at various intervals. Used primarily as experimental procedures, these paradigms have furthered our understanding of learning and plasticity.

Another paradigm to deliver TMS pulses is repetitive TMS (rTMS) in which trains of multiple pulses are applied at particular frequencies. This kind of stimulation has more prolonged effects on brain circuits, and the aftereffects depend on the intensity, frequency, number of stimulations, and the state of contraction/ relaxation. The two major effects are a facilitatory one, with frequencies $>$ 1 Hz [36] and inhibitory effects with frequencies $<$ 1 Hz [37,38]. The duration of such effects depends mainly on the number of stimuli administered. In addition, a newly introduced paradigm, the so-called theta-burst stimulation (TBS) [39], uses short bursts of low intensity (80% of MT), high-frequency (50 Hz) pulses repeated at 5 Hz frequency, analogous to the EEG theta rhythm, which is thought to exert an effect on the synapses between interneurons responsible for the indirect spinal I1 wave and corticospinal neurons [40]. Different theta-burst patterns, i.e., intermittent vs. continuous, yield opposite effects on the stimulated cortex [39,41,42]. A recent study by McAllister et al. [43] selectively targeted intracortical inhibitory networks for modulation by low-intensity TBS, but the responses appear to depend upon the particular paradigm chosen [43].

Safety issues have been raised regarding the use of rTMS in both healthy individuals and epileptic patients. These concerns have been addressed in recent guidelines by Rossi et al. [44]. In their review of all reported cases of TMS seizure induction, they concluded that the risk is certainly very low for both the rTMS paradigm and the single-pulse paradigm (16 cases out of 143 studies) [44], although rTMS seems to harbor a potentially

Table 1
Principal TMS parameters for monitoring antiepileptic drug-induced changes in cortical excitability

| Parameter | Protocol | Neurophysiological mechanism | Modulating AED |
|---|---|---|---|
| Motor threshold (MT) | Single-pulse stimulation | Corticomotor neuron membrane excitability | Increased by sodium channel antagonists (e.g., PHT, CBZ) |
| Short-interval intracortical inhibition (SICI) | Paired-pulse stimulation: conditioning stimulus precedes test stimulus by 1–5 ms | GABAA-mediated inhibition | Increased by GABAA agonists |
| Intracortical facilitation (ICF) | Paired-pulse stimulation: conditioning stimulus precedes test stimulus by 6–20 ms | Glutamate-mediated Excitation | Decreased by GABAA agonists |
| Long-interval intracortical inhibition (LICI) | Paired-pulse stimulation: conditioning stimulus precedes test stimulus by 50–300 ms | GABAB-mediated inhibition | Increased by GABAB agonists |
| Cortical silent period (CSP) | Single-pulse stimulation: measures lapse in voluntary EMG activity after TMS | GABAB-mediated inhibition | Increased by GABAB agonists; decreased by GABAA agonists |

higher risk. A critical issue is the possible interaction of hazardous drugs, i.e., drugs that lower the seizure threshold, since the majority of r-TMS-induced seizure events occurred in individuals under therapy with such drugs.

Given the overall safety of TMS, its potential clinical application to epilepsy has advanced translational research into how to combine TMS with other techniques in a multimodality approach or to study behavioral manifestations.

Recent advances in TMS-EEG co-registration in humans and animals may enhance clinical and translational TMS/rTMS applications [45–59]. At the time of this writing, TMS-EEG is still mostly used in human nonclinical studies of cortical excitability and connectivity, and has not been extensively applied in patient populations or animal disease models.

Presently, TMS-EEG holds promise as an experimental method to noninvasively test seizure detection algorithms in combination with abortive stimulation patterns, perhaps as a tool that will aid in designing other forms of responsive cortical stimulation.

Although EEG-guided TMS has not yet been applied in clinical practice, the potential for using real-time EEG to direct TMS/rTMS is suggested by several reports which demonstrate that the EEG state predicts the cortical response to TMS. For example, MEP amplitudes correlate with EEG power in the alpha and beta frequency range recorded with electrodes positioned over the motor cortex [60]. This preferential state-dependent response to TMS in healthy volunteers suggests that studies of EEG guidance in patient populations is warranted and perhaps well-suited for epilepsy, where the ictal and interictal EEG states are often easily distinguished by visual inspection or an automated EEG algorithm. On a finer temporal scale, TMS-EEG may enable time-locking of TMS to a specific phase of an underlying EEG signal while testing the time course of EEG reactivity to the magnetic pulse [46,47, 61,62]. Recently, time-frequency analysis has led to a better understanding of the effect of brain stimulation on brain oscillatory rhythms, with a rapid desynchronization of activity in the alpha and beta bands and a rapid synchronization of delta and theta activity [63].

TMS-compatible scalp EEG electrodes and electronic components designed to minimize TMS artifacts are relatively inexpensive and can be adapted to most existing clinical and research EEG setups for real-time EEG recording during TMS/rTMS [53]. The possible applications of TMS-EEG recording include diagnostic measurement of cortical excitability, real-time monitoring for epileptiform EEG activity during rTMS in vulnerable populations, and designing therapeutic rTMS protocols.

## 2. TMS and antiepileptic drugs

Our understanding of the TMS mode of action derives, beyond the basic neurophysiological principles underpinning it, from the study of the interaction of drugs with a known mechanism of action and TMS parameters. Well-defined TMS measures are helpful tools to define the mode of action of a study drug. Applying this knowledge to a pathologically excitable brain and observing the drug-induced modulations could point to an underlying dysfunction.

As far as this review is concerned, there is a wealth of data on the effects of antiepileptic drugs (AEDs) on TMS parameters.

Motor threshold, depending on cortico-cortical axon excitability [64], is largely influenced by voltage-gated sodium channels blockers, such as carbamazepine (CBZ), phenytoin (PHT), and lamotrigine (LTG), that elevate the MT [13,65–69]. Recently, Solinas et al. suggested that a modulatory effect on high-threshold calcium currents and perhaps on potassium channels could also affect the MT, after observing its increase after acute levetiracetam (LEV) administration [70,71]. Sulthiame, a carbonic anhydrase inhibitor, similarly increases the MT, plausibly operating on the same sodium voltage-gated channels [72]. Conversely, NMDA antagonists such as ketamine lower it by indirectly increasing AMPA-mediated transmission [73].

Motor evoked potential amplitude, which at high stimulus intensity appears to be generated through a chain of cortical excitatory interneurons [74,75], is mainly modulated by neurotransmitters (glutamate, GABA) and neurotransmission modulators (DA, NE, 5-HT, Ach). Among the AEDs, benzodiazepines, as GABAA receptor modulators, decrease the MEP amplitude [76,77], as does zonisamide [78].

The cortical silent period (CSP) is postulated to depend on a long-lasting inhibition of the motor cortex [79], and GABAB receptors [80,81] seem to modulate the last part of the CSP through supraspinal structures [82,83]. This mechanism was proposed based on the observation that tiagabine [80] and pregabalin [84] lengthen CSP. An indirect effect on the same receptor is believed to explain the effect induced by levetiracetam [70].

Studies investigating the effect of GABAA have produced discordant results: Ziemann [13,85] described an increase after the administration of lorazepam and ethanol, probably due to induced attentional deficits, while a decrease after diazepam administration was observed by Inghilleri et al., Palmieri et al. and Ilic et al. [76,77,86].

Paired-pulse stimulation parameters include SICI, ICF and SICF.

SICI is thought to be modulated by GABAA agonists such as valproate (VPA) [87] and lorazepam [13,65], which enhance its effect presumably by potentiating the inhibitory postsynaptic potential (IPSP) induced by the first subthreshold stimulus [2,9,12,86,88]. In fact, the observation by Werhahn et al. [80] that tiagabine decreases SICI is in line with this finding on the basis of drug auto-inhibition of inhibitory neurons, and an analogous mechanism has also been proposed for pregabalin [84].

The physiology of ICF is unclear; it has been postulated that its effects on the motor cortex excitatory circuits [2] are mediated by excitatory neurotransmitters such as NMDA receptors [89] and a weak inhibition mediated by GABAA receptors [90]. Based on these findings, ICF decreases with GABAA agonists such as the benzodiazepines [2,76], and with NMDA agonists [20,91].

Finally, SICF is thought to act on the excitatory interneurons that are depolarized by the first pulse but have not yet fired. Thus, GABAA drugs (BDZ) or increased GABA amounts (gabapentin [GBP]) reduce SICF [86,88,92], given that the first pulse elicits GABAA-dependent IPSPs.

Several studies focused on the interaction of AEDs and repetitive TMS (rTMS) [93,94]. Given that high-frequency rTMS (5Hz) progressively increases the size of MEPs and the duration of the CSP, the observation that CBZ, GBP and topiramate (TPM) abolish the rTMS facilitation of MEPs but do not act on the CSP leads to postulate a selective effect of rTMS on excitatory intracortical interneurons, probably by interfering with rTMS-induced synaptic potentiation. A study by Palermo et al. [95] in migraine patients, evaluating the phosphene threshold with 1 Hz rTMS, suggested a GABAergic modulatory mechanism of VPA that restored inhibitory intracortical circuits.

## 3. TMS and anesthetics

Numerous studies have investigated intraoperative anesthetics and their effects on neurophysiological parameters monitored during surgery. These are mainly MEPs and sensory evoked potentials (SEPs). In the surgical setting, the former are evoked mostly by transcranial electrical stimulation (TES) and not standard TMS, given that the TES device is more manageable – less bulky and with electrodes fixed on the head of the patient. In such settings, MEP evaluation involves amplitude modifications of the potential; and the influence on these parameters of anesthetics is important for correctly evaluating the observed response.

To our knowledge, only one experimental study by Ferrarelli et al. [96] investigated the effect of TMS and midazolam-induced anesthesia, describing a decrease in cortical effective connectivity in comparison to the wake state in healthy individuals. The decreased effective cortical connectivity was described by the authors as a restriction of the cortical areas where the TMS-induced waves were recorded, and a modulation of its duration and intensity. Interestingly, another paper by the same group [97] showed a similar pattern, neurophysiologically bridging the two states.

## 4. TMS in epilepsy: A rationale for neuromodulation

An attractive role for TMS-EEG in epilepsy may be that of a neurophysiological stressor to provoke epileptiform activity in a vulnerable cortical region. Induction of epileptiform discharges by TMS in human subjects has been demonstrated, although very early studies suggested that TMS was no more likely to activate a seizure focus on EEG than was hyperventilation in epileptic patients [98–100]. However, more recently, Valentin et al. [101] applied single-pulse TMS-EEG to patients with focal epilepsy and to a group of healthy controls. They identified two broad categories of EEG evoked response: an early (< 100 ms) slow-wave response and a late (100–1000 ms) response which was either epileptiform in morphology (resembling a sharp wave or spike) or was characterized by rhythmic EEG activity.

An extension of these data, showing epileptiform activity provoked by TMS-EEG and localized to one hemisphere, is toward a more detailed seizure focus localization, as is necessary in cases where surgical seizure focus resection is considered.

The rationale for TMS as a therapeutic tool is based on the fact that repetitive TMS (rTMS) can produce effects that outlast the application of a train of stimuli by minutes or hours. Low-frequency rTMS (< 1 Hz) reduces cortical excitability, as evidenced by a longer duration of the cortical silent period [102] and reduced motor-evoked potential amplitudes [103]. In contrast, higher frequencies (> 5 Hz) enhance cortical excitability. These effects resemble long-term depression (LTD) and long-term potentiation (LTP), two forms of synaptic plasticity elicited in animal models by low- and high-frequency electrical stimulation of cortical circuitry.

In epilepsy, it is the inhibitory effect of low (< 1 Hz) rTMS that is most widely used to suppress seizures, with encouraging results in open-label trials [104–106]. Yet, results from placebo-controlled trials are mixed, with one trial demonstrating a reduction in seizures and improvement of (off-line) EEG [107], and two others showing insignificant clinical improvement, or improvement of the EEG without a significant reduction in seizures [108–110]. Finally, Brodbeck et al. [111] found no decrease in seizure frequency after rTMS [111]. Among the factors contributing to the inconsistent findings in antiepileptic rTMS trials may be the difficulty in selecting an appropriate intensity of extramotor TMS, i.e., stimulation output intensity outside the motor cortex. Another limitation is the shallow penetration effect of the induced electromagnetic field that stimulates only the superficial cortical layers, but is unable to affect the functioning and discharge of deep subcortical studies. This consideration may explain the better results observed in patients with neocortical foci [112], and the overall better results of other brain stimulation techniques. A recently concluded multicenter, double-blind, randomized trial [113] showed over a 3-year follow-up a 56% reduction in seizures in patients with medically refractory partial or secondary generalized seizures implanted with electrodes stimulationing the anterior nucleus of thalamus. Other deep brain stimulation targets are: the centromedian thalamus, with outcomes depending on the seizure type in a small series by Velasco et al. [114] or no effect at all [115,116]; the subthalamic nucleus, with a significant reduction in seizure frequency, although results are based on small samples [117–121]; the cerebellum, with little [122] or no improvement [123,124]; the posterior hypothalamus, usually stimulated in cluster headache, but proved efficacious also in two epileptic patients by Franzini et al. [125]; the head of the caudate [126–128]; and the hippocampus, which is mainly stimulated in mesial temporal epilepsy refractory or not amenable to surgery, or in cases of dual pathology, that yielded discordant results [129–131]. Anecdotally, the corpusm callosum [132] and the locus ceruleus [133] have also been stimulated with little or no benefit. Another stimulation method is vagal nerve stimulation (VNS) that showed an effect comparable to those seen in trials of new antiepileptic drugs for patients with refractory complex partial seizures, although selection criteria seem to be fundamental in the outcome [134–143].

The possibility to combine TMS and EEG could improve not only the sensitivity of the TMS method but also the efficacy of neurostimulation. In the clinical setting, where the majority of TMS/rTMS work has focused on the interictal state, TMS-EEG can be applied in the ictal state to identify real-time EEG changes induced by rTMS. TMS-EEG also detects seizure improvement or exacerbation, making it a valuable clinical tool for everyday practice. TMS-EEG was recently applied in a small series of epilepsia partialis continua (EPC) to detect seizure suppression and to exclude seizure exacerbation during rTMS in animal models [144–146] and in humans [147–150]. Encouragingly, rTMS did not induce seizures, while seizure suppression was detected in some instances. Similar TMS-EEG methods may be of use to monitor for evoked epileptiform activity when rTMS is administered to treat non-epileptic symptoms such as mood disorder, motor dysfunction or chronic pain in seizure-prone patients, such as those with recent stroke, neurodegenerative disease or underlying epilepsy, or in patients on seizure threshold-lowering drugs.

A future field of application of TMS-EEG techniques is related to the emerging concept of systemic epilepsy, moving a step forward from the concept of an epileptogenic area to the idea of an overall pathological circuitry in the epileptic brain. This concept holds true particularly for generalized epilepsies, based on observations by Manganotti et al. [33,34] and Del Felice et al. [151] that an overall higher excitability is elicited by TMS in juvenile myoclonic epilepsy (JME).

## 5. The study of consciousness: The perturbational approach

In theoretical neuroscience, consciousness does not correspond to activity level, access to sensory inputs or neural synchronization *per se*, but rather consists of the ability of different areas of the thalamocortical system to interact causally with each other to form an integrated whole. The information integration theory of consciousness (IITC) argues that consciousness is an integrated information state and that the brain should be able to generate consciousness to the extent that it has a large series of available states (information), yet it cannot be decomposed into a collection of causally independent subsystems (integration) [152,153] To evaluate the brain's ability to integrate information across distributed cortical regions, it may not be sufficient to observe the brain in action. Instead, it could be useful to employ a perturbational approach and examine to what extent different regions of the thalamocortical system can interact causally (integration) and produce specific responses (information). With TMS-EEG the immediate reaction of the entire thalamocortical system can be recorded to controlled perturbations of different cortical areas. Most recent studies have used sleep as a model of unconsciousness in which TMS-EEG is appliedto detect changes in the ability of the thalamocortical system to integrate information when the level of consciousness fluctuates across the sleep-wake cycle.

Massimini et al. [154] showed that in normal subjects TMS triggering of slow waves reveals intrinsic bistability in the thalamocortical networks during non-rapid eye movement (NREM) sleep. Moreover, evoked slow waves lead to a deepening of sleep and an increase in EEG slow-wave activity (0.5–4.5 Hz), which is thought to play a role in brain restoration and memory consolidation. It is well known that during much of sleep cortical neurons undergo near-synchronous slow oscillation cycles in membrane potential, which give rise to the largest spontaneous waves observed on the normal electroencephalogram [155]. Slow oscillations underlie characteristic features of the sleep EEG, such as slow waves and spindles.

When combined, TMS and EEG provide a means to study the reactivity of cortical regions in the intact brain; also the reactivity of non-motor cortical areas related with higher-order functions is now appreciable. A recent epochal finding in cortical reactivity is that neuronal activation is induced by remarkably low stimulation intensities [155]. This knowledge is significant when optimizing experimental setups for maximal patient safety. Stimulation of different cortical areas evokes different patterns of remote EEG activity, confirming the viability of TMS–EEG for the study of cortico-cortical connections. In this review, we discuss these and other notable findings related to TMS–EEG [156]. Under investigation are different models of loss of consciousness such as deep anesthesia in normal subjects [157] and vegetative states [158,159].

## 6. Consciousness and epilepsy

Epilepsy can provide a study model for investigating the dynamic modifications of consciousness. In epilepsy, brain-state changes that may occur long before seizure onset, and potentially triggering a seizure, are not well understood. The estimation of dynamic changes in brain state associated with disease or stimulation is relevant for both diagnostic purposes and optimizing therapeutic stimulation.

In epilepsy, sleep and sleep deprivation are two conditions that dynamically modulate brain activity, with important clinical consequences. TMS has been used to further our understanding of the effects of sleep and sleep deprivation on cortical excitability in healthy and epileptic patients. In healthy subjects, sleep deprivation produces a mild decrease in cortical excitability during nighttime that is probably related to drowsiness, although no differences in TMS values have been observed either before or after sleep deprivation [33]. It has been reported that corticospinal fibers are normally activated by magnetic stimulation, while motor excitability is decreased during different sleep stages [17, 33]. In contrast, other authors [160] have observed that in normal subjects, 24 hours of sleep deprivation produce an increase in motor excitability as studied by TMS in two sessions, namely, before and after sleep deprivation. The different effects on motor excitability of sleep deprivation could be due to the differences in study methods and objectives. While earlier studies were performed using TMS alone, an important advance has been made with the application of TMS-EEG- co-registration to the effects of sleep and sleep deprivation in healthy subjects [151]. The authors observed a significant effect of sleep deprivation on cortical excitability, defined as an amplitude increase of TMS evoked potentials (TEPs). Nevertheless, the sensitivity of TMS, and to a major extent of TMS-EEG co-registration, to sleep deprivation has introduced a new feature in clinical research as it allows the generation of hypotheses to account for the changes in motor excitability in epileptic patients after activation tests commonly used in clinical practice (i.e., sleep deprivation). Following this line of research, the study of motor excitability in epileptic patients after sleep deprivation demonstrated a reduction in SICI in benign myoclonic epilepsy patients compared to healthy subjects. The paired-pulse method allows measurement of so-called short latency intracortical inhibition (SICI) and short latency intracortical facilitation (SICF) at short interstimulus intervals (ISIs) (1–25 msec) [2]. Intracortical inhibition and facilitation are thought to reflect the excitability of separate populations of interneurons intrinsic to the cortical motor area. In fact, a reduction in cortical inhibition has been observed in different forms of epilepsy, including progressive myoclonic epilepsy [34,35,161], juvenile myoclonic epilepsy (JME) [33, 162], generalized epilepsy [163], and partial epilepsy [164,165]. All these studies have been performed in epileptic patients when awake after normal sleep. A recent TMS-EEG study in JME patients after partial sleep deprivation described an impressive increase in cortical excitability, as measured by amplitude augmentation of TEP [151].

Information on sleep brain-states in epilepsy, based on either motor excitability tested by TMS or EEG reactivity to TMS perturbation is lacking. Salih et al. [166] investigated motor cortical excitability with paired TMS without a TMS-EEG system during NREM sleep in epileptic patients and showed an increase in intracortical excitability during sleep with a pattern opposite that observed in normal subjects. Del Felice et al. [151] observed an increase in excitability during NREM sleep (mainly S2) in JME patients but not in healthy controls. Similarly, we have only few case reports on the effect of TMS delivered inside a paroxysmal activity, with the stimulus evoked by either peripheral stimulation [32] or spontaneous [167]. Some studies used a TMS-EEG system to avoid delivering TMS during spike and wave activity and to test motor excitability outside paroxysmal activity [168].

In generalized epilepsy with the typical burst of 3 Hz spikes and waves, there are usually transitory periods of loss of consciousness without more complex epileptic phenomena. In other forms of epilepsy, such as Janz syndrome epilepsy or focal epilepsy, diverse and distinct episodes of loss of consciousness can occur. What remains to be discovered is the possible causal relationship between different levels of cortical excitability and its prevalent cortical localization, and the degree of consciousness impairment.

## 7. Future directions

TMS-EEG allows the investigation of brain excitability correlated to paroxysmal activity and to episodes of loss of consciousness in epileptic patients. The possibility to deliver a magnetic pulse before or during symptomatic or asymptomatic discharges monitored by TMS-EEG will be a further step toward understanding the level of integration of the thalamocortical system. The study of sleep in epilepsy by brain stimulation is an open field where the introduction of the perturbation method during sleep could add important information on the integration and connectivity of cerebral circuitry in epilepsy. The study of focal seizures and focal paroxysms by means of TMS can drive important advances in the clinical setting, where translational methods are evolving.

## 8. Conclusions

At present, the clinical role of TMS-EEG in epilepsy is uncertain, and a discussion of its applications in the clinical arena is necessarily speculative. However, recent data suggest that exploration in patient populations is warranted, and the adaptation of TMS-EEG to translational research may help to clarify its role as a diagnostic or therapeutic tool. Especially attractive in clinical epilepsy are the prospects for TMS-EEG as a way to test regional cortical excitability, to more accurately detect an activation thresholds for the extramotor cortex and to determine an anticonvulsive effect or a proconvulsive side effect of repetitive stimulation. As the necessary technology for TMS-EEG is now widely available, meaningful clinical and translational trials in the near future seem likely.
